# JunctionViewer: customizable annotation software for repeat-rich genomic regions

**DOI:** 10.1186/1471-2105-11-23

**Published:** 2010-01-12

**Authors:** Thomas K Wolfgruber, Gernot G Presting

**Affiliations:** 1 Department of Molecular Biosciences and Bioengineering, University of Hawai'i at Mânoa, 1955 East-West Road, Ag Science Bldg. Rm 218, Honolulu, HI 96822, USA

## Abstract

**Background:**

Repeat-rich regions such as centromeres receive less attention than their gene-rich euchromatic counterparts because the former are difficult to assemble and analyze. Our objectives were to 1) map all ten centromeres onto the maize genetic map and 2) characterize the sequence features of maize centromeres, each of which spans several megabases of highly repetitive DNA. Repetitive sequences can be mapped using special molecular markers that are based on PCR with primers designed from two unique "repeat junctions". Efficient screening of large amounts of maize genome sequence data for repeat junctions, as well as key centromere sequence features required the development of specific annotation software.

**Results:**

We developed JunctionViewer to automate the process of identifying and differentiating closely related centromere repeats and repeat junctions, and to generate graphical displays of these and other features within centromeric sequences. JunctionViewer generates NCBI BLAST, WU-BLAST, cross_match and MUMmer alignments, and displays the optimal alignments and additional annotation data as concise graphical representations that can be viewed directly through the graphical interface or as PostScript^® ^output.

This software enabled us to quickly characterize millions of nucleotides of newly sequenced DNA ranging in size from single reads to assembled BACs and megabase-sized pseudochromosome regions. It expedited the process of generating repeat junction markers that were subsequently used to anchor all 10 centromeres to the maize map. It also enabled us to efficiently identify key features in large genomic regions, providing insight into the arrangement and evolution of maize centromeric DNA.

**Conclusions:**

JunctionViewer will be useful to scientists who wish to automatically generate concise graphical summaries of repeat sequences. It is particularly valuable for those needing to efficiently identify unique repeat junctions. The scalability and ability to customize homology search parameters for different classes of closely related repeat sequences make this software ideal for recurring annotation (e.g., genome projects that are in progress) of genomic regions that contain well-defined repeats, such as those in centromeres. Although originally customized for maize centromere sequence, we anticipate this software to facilitate the analysis of centromere and other repeat-rich regions in other organisms.

## Background

### Large-scale Discovery of Repeat Junction Markers

Centromeres are chromosomal regions that are essential for chromosome segregation during cell division. Maize (*Zea mays*) centromeres span several megabases each and consist predominantly of repetitive DNA that is enriched for the tandemly arranged CentC satellite sequences as well as certain subfamilies of the retrotransposon family named "centromeric retrotransposons of maize" (CRM) [1-4]. CRM1, CRM2 and CRM3, as well as the non-autonomous CRM3 element CentA, can insert into each other, as well as into CentC satellite repeats and other DNA sequences found in centromeres. Although these elements insert preferentially into active centromeres, they do not appear to target specific sequences.

Due to their centromere-targeting integration mechanism, CRM elements are excellent cytogenetic markers for centromeres [1,2,5,6]. However, genetic mapping requires the use of single copy molecular markers. The estimated 500 copies of CRM elements are very similar to each other, but each retrotransposon insertion creates two "repeat junctions" that, due to the fact that retrotransposon insertion is not sequence-specific, have the potential to be unique even if the target sequence itself is a high-copy retrotransposon. Any given repeat junction can be used in combination with the repeat junction of another element inserted nearby to create a single-copy marker [7]. Mapping the highly repetitive CRM elements, and thus the centromeres, using the "junction marker" approach therefore required the efficient identification of repeat junctions no more than 2.5 kb apart in maize genomic DNA sequence.

An added complication of using CRM elements for repeat junction identification is that several CRM subfamilies were discovered to contain two "parental" variants with about 80% identity that, in the case of CRM1, form at least 5 different recombinants with different periods of activity [8]. These recombinants contain up to 5 recombination breakpoints that separate segments that are close to 100% identical to one parent or the other, and would appear as repeat junctions if they were annotated using only the parental variants.

### Graphic Display of Sequence Annotation

In addition to the centromere-specific repeat junctions, we wanted to display other important features in our graphical representation of centromere sequences. LTR retrotransposons, which represent >75% of the maize genome [9], are named for their long terminal repeats, which are identical at the time of insertion and can be used to date the insertion time of each element [10]. We used MUMmer [11] to identify the LTRs of unknown retrotransposons. Although maize centromeres consist primarily of centromere-specific and other repeat elements, we expected them to also contain genes and organellar sequences based on the previously characterized rice centromeres [12] and maize fluorescent *in situ *hybridization results [13], respectively. Furthermore, centromeric DNA is marked epigenetically by the centromeric histone H3 variant CENH3, and antibodies against this histone have been used successfully to immunoprecipitate centromeric DNA in maize [5]. Sequencing and mapping of such DNA fragments is an efficient means for delineating the border of the functional centromeres [9,14,15].

All of these features are detectable by sequence homology using widely available software such as NCBI BLAST [16], WU-BLAST http://blast.wustl.edu/, cross_match http://www.phrap.org/, and MUMmer. Since all CRM elements are homologous to each other as well as to other retrotransposons at some level, we needed the ability to use different alignment parameters for different sequence features depending on the feature length and the level at which we wanted to distinguish related sequences (e.g., CRM1 recombinants).

Sequence annotation is often displayed in a stacked format (e.g., in the genome browser Ensembl http://www.ensembl.org) with the intent of letting the user decide which features are important or real. The DAWGPAWS scripts [17] run a variety of sequence similarity and gene prediction programs for finding genes and repeat elements, the output files of which are converted in subsequent steps into the gff format that can be read by a genome browser (e.g., GBrowse [18]), but again the voluminous annotation is displayed in a stacked format. Although the stacked format can be very useful for displaying variants of a given genomic region (e.g., differentially spliced ESTs or cDNAs), this is not desirable for the display of repeats. Instead, it was critically important that our software be able to select the single repeat subclass present at a particular genomic locus and display it in an easily comprehensible manner.

JunctionViewer was developed to automate the process of discovering, filtering, and visualizing key features of the recently sequenced DNA of maize centromeres. It allowed us to expediently sift through and understand these complex sequences, significantly decreasing the time required to analyze the centromere sequences of maize [15]. Automatic reannotation of evolving genome sequences as they passed through the stages of finishing, as well as the ability to add relevant novel repeat classes as they were discovered in the emerging sequence, are two key applications of this software.

## Implementation

JunctionViewer (2.0) is a Perl script using the Tk module to manage its GUI and the BioPerl module to parse biological data files [see Additional file 1]. These modules can be obtained through CPAN http://www.cpan.org/.

Input to the script is provided by a single text file, which defines all user parameters. If no file is given the script generates a template file with comments describing argument fields.

The parameters file is composed of six sections: 1) General parameters (e.g., where the query sequence file is located), 2) NCBI BLAST parameters for each subject sequence database (e.g., *E*-value filter), 3) WU-BLAST parameters for each subject sequence database, 4) color schemes for displaying BLAST database sequences, 5) color assignments for cross_match subject sequence databases, and 6) custom annotation charts parameters (i.e., which charts will overlap others and in what color each chart will be drawn).

**Figure 1:**
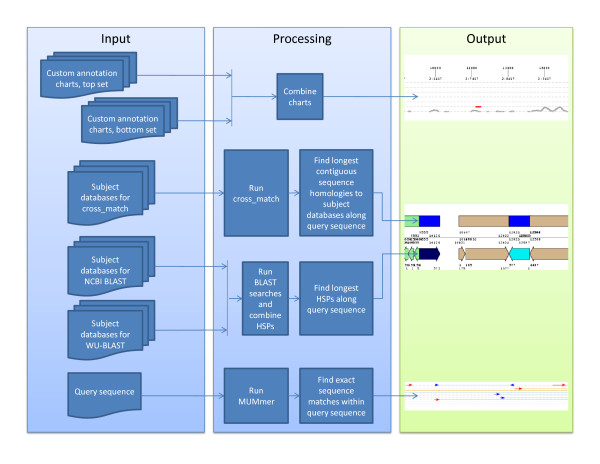
**Data flow through JunctionViewer 2.0**. This figure illustrates how JunctionViewer 2.0 transforms input documents into a graphical representation of DNA sequence features within a query sequence. The query sequence represented here is BAC CH201-530C10 (GenBank accession AC184133.3) (Figure 2).

The script runs nucleotide NCBI BLAST, WU-BLAST, cross_match, and MUMmer, and combines these results, as well as custom annotation data (in the form of a numerical value per query nucleotide) into one graphical display per query sequence (see data flow in Figure 1).

Tick marks at the top of each display indicate sequence locations at 1 kb intervals. If 100 contiguous Ns are found within the query sequence (representing gaps within sequence assemblies), a grey vertical bar is drawn. A secondary value below each tick mark represents the number of nucleotides relative to the end coordinate of the last gap.

Below the query coordinates, custom data is represented as overlapping charts in two chart sets. Different y-axes (scale/maximum) may be used for each of the two chart sets (foreground and background). A numerical y-value can be assigned to each nucleotide on each chart, and a different color can be assigned to each chart. The y-axis is automatically extended to the largest value in a given chart.

In the current implementation, JunctionViewer displays homology data in separate cross_match, BLAST and MUMmer panels. Under the charts are displayed sequence homologies based on cross_match results that are represented as filled boxes. Longer contiguously masked sequences are drawn over and eliminate shorter ones, unless the overlap is within a user-defined allowance (set in the parameters file). Each masking database may be assigned a color. Query sequence coordinates of the homologous sequences are also indicated.

BLAST alignments, shown as large filled arrows that indicate HSP orientation, are drawn below the cross_match results. Start and stop coordinates are given for both query and subject sequences. Parameters for each subject database are defined individually. All NCBI and WU BLAST HSPs are combined and competed, i.e., longer HSPs eliminate shorter overlapping ones if they correspond to sequences from different databases, unless the overlap is within a user-defined allowance. If HSPs are generated from sequences within the same database, longer HSPs will overlap but not eliminate shorter ones. This aids in the display of tandem repeats. Each sequence in a BLAST database may be assigned a color. Additionally, subsequence positions within subject sequences may be assigned different colors.

At the bottom of the display, exact sequence matches identified with MUMmer (and user-defined minimum match length), are drawn as thin line arrows. Solid lines indicate exactly matching sequence regions and are connected by dashed lines. Direct and indirect repeats are colored red and blue, respectively. Lines can be drawn on a fixed number of levels (currently set in the main program to 30). The longest matches are drawn first on the top level, and smaller matches that overlap are drawn on lower levels until the number of levels is exceeded.

If a FingerPrinted Contig (FPC [19]) assembly project exists for the query sequences, the project file (.fpc) can be provided as an argument and the script will label and sort the query sequences in the GUI and name PostScript output files based on their positions in the FPC project. This is useful for labeling individual annotated BACs of a set (e.g., from a single FPC contig), which facilitates subsequent arrangement in the correct order (e.g., if they are to be printed out on paper).

Results can be viewed through the JunctionViewer GUI or from PostScript file results. Within the GUI, the number of nucleotides displayed per pixel can be modified, thus changing the zoom of the resulting image. PostScript files are created automatically each time that a given sequence is visualized in the GUI by either highlighting the ID and pressing the "Display single selection" button, or by pressing the "Process all displays" button. PostScript files can be converted into other graphic file formats and imported into Microsoft^® ^PowerPoint^®^, enabling the display of entire centromeres or other regions spanning 5-10 megabases. We routinely use JunctionViewer to analyze 210 kb genomic fragments that overlap by 10 kb to ensure that features of 10 kb or less (e.g., LTRs) are drawn completely at least once.

On an AMD Athlon™ 64 Processor 3500+ (2.2 GHz) with 4 Gb 166 MHz DDR3 RAM running Fedora™ Linux, it took 4 minutes 43 seconds to generate graphical displays of the two 210 kb centromeric chromosome 5 sequences shown below. Maximum RAM memory usage is 205 Mb and output files, including PostScript output, total 56 Mb.

JunctionViewer 2.0 can be setup and used in four steps: 1) the installation of supporting software (e.g., Perl), 2) populating a parameters file, 3) revising the parameters file as necessary after testing with previously characterized sequences, and 4) using the refined parameters file to automatically annotate uncharacterized sequences [see Additional file 2].

Although defining all parameters for each repeat and algorithm takes some time during the initial setup for each new organism, this up-front cost is quickly compensated by the ease with which large tracts of genomic DNA can subsequently be analyzed and reanalyzed as genome sequence is improved.

## Results and Discussion

Prior to our discovery of recombinant CRM subtypes, we displayed sequence features in shotgun reads (~700 nt) using a JunctionViewer 1.0 script that generated stacked annotations. This was effective for screening short reads for junctions using a small set of distinctly different repeats, i.e., CRM1, CRM2 and CentC. The coordinates determined by the software were subsequently used to manually design primers across junctions, but alternatively the JunctionViewer output could be used to generate primer sequences automatically.

The Maize Genome Sequencing Consortium [9] produced large (~160 kb) assembled BAC sequences that made finding pairs of nearby junction sites easier, but also resulted in JunctionViewer 1.0 displays becoming cluttered and difficult to interpret [see Additional file 3]. Novel recombinant CRM elements were discovered during the sequencing phase, and these needed to be differentiated in our graphical displays. At the same time we needed to visualize a number of other features, including gene homologies and CENH3 associated sequences.

We therefore developed JunctionViewer 2.0 to automatically generate compact graphical representations of only the best fitting annotations for the larger and more complex BAC sequences. With this software we were able to distinguish and visualize not only the 3 centromere-enriched subfamilies of CRM [4] but also five major subgroups of CRM1 [8], as well as other repeat types and gene coding sequences as condensed graphical representations. The centromeric repeats we selected for representation were LTRs from 5 subfamilies of CRM1 (A, B/R1, R2, R3 and R4/R5), as well as CRM2, and CRM3. Polyprotein coding sequences (CDSs) of these three CRMs were displayed in the same color. Differently colored CRM LTRs flanking CDSs were used to identify the CRM element types. We chose not to draw the UTRs since these highly variable and low-complexity regions are difficult to consistently detect. We also displayed LTRs and CDS of the non-autonomous centromeric retrotransposon CentA, the centromeric tandem repeat CentC, non-centromeric "maize repeats" [20], "maize genes", "maize organelle" sequences, and "rice genes" [see Additional file 4]. We did this by adjusting the parameters used for each of the sequence databases.

We developed our JunctionViewer parameters by estimating the score obtained for a near-perfect match to each of the different sequence databases and subsequent testing of those parameters using a set of characterized sequences (e.g., known CRM subtypes). For example, we set the score-equivalence threshold (S) of WU-BLAST to 10% less than a perfect complete match in order to distinguish CRM2 and CRM3 LTRs. The detailed process of refining the parameters to achieve the desired sensitivity, specificity and resolving power will vary - in some cases we had to fine-tune the parameters until the features we wanted to visualize were properly displayed by the software. We set MUMmer to report exact sequence matches >= 100 nt.

We used the top section of the JunctionViewer 2.0 display to plot the coverage of CENH3-associated centromeric sequences that had been obtained by chromatin immunoprecipitation followed by pyrosequencing (ChIP-Seq). Three categories of pyrosequencing reads were created based on the number of occurrences in the genome sequence, and visualized using 3 graphs and two different y axes. Plots were generated based on the number of reads covering a given nucleotide, e.g., a nucleotide covered by two reads was assigned a value of 2.

**Figure 2:**
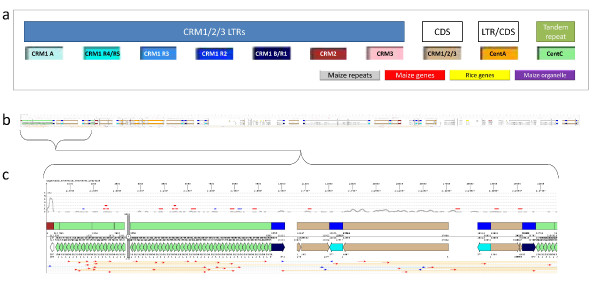
**JunctionViewer 2.0 display of a BAC sequence**. (a) Sequences annotated include: LTRs from 5 subfamilies of CRM1 (different shades of blue) [8], CRM2 (maroon), and CRM3 (pink) as well CDSs of CRM1/CRM2/CRM3 (tan), CentA LTR and CDS (orange), and tandem repeat CentC (green). For clarity, all CRM CDSs are drawn in the same color - the subfamily and recombinant subtype of each CRM is identified by the LTR. UTRs are not displayed, since these are highly variable regions including long stretches of homopolymers. Our annotations also included non-centromeric "maize repeats" (grey), "maize genes" (red), "maize organelle" (purple), and "rice genes" (yellow) homologous sequences [for details please see Additional file 4]. (b) Birds-eye view of the complete BAC CH201-530C10/AC184133.3 (labeled c0530C10 in the image as named in FPC) showing the complex arrangement of CentC and CRM sequences. Two overlapping chart sets above the BLAST/cross_match homologies, use different y-axes that indicate the number of anti-CENH3 ChIP-Seq reads covering each nucleotide. Red and blue datapoints represent coverage by reads that match one or two BACs, once or any number of times within the BAC, respectively. Grey points are plotted in the background graph using a different y-axis and represent coverage by reads matching any number of BACs any number of times within a BAC. Thus, the grey charts indicate the general degree of association of a given sequence class (e.g., CRM element) with the centromere protein CENH3, while red and blue charts highlight sequence regions that are specifically bound to CENH3. At the bottom of the display, thin red and blue arrows indicate >= 100 nt exactly matching within the query. (c) Close-up of a BAC section showing nested insertions consisting of a CRM1 B element insertion into a CentC array, followed by insertion of a CRM1 R4 or R5 element into the CRM1 B element, moving the CentC sequences even further apart.

For BACs, which can overlap considerably with neighboring BACs, pyrosequence reads that mapped to one or two BACs in a set representing the genome (15,101 BACs sequenced by the Maize Genome Sequencing Consortium, downloaded from GenBank on 25 January 2008) were used to create two charts sharing one y-axis. The first chart displayed nucleotide coverage for reads that mapped to only one location within the BAC. The second displayed coverage for reads that mapped any number of times within that BAC. A third chart was used to display coverage for reads that mapped any number of times to any number of BACs in the genome set. This third chart was plotted in the same display region as the first two but on a separate y-axis under the others (Figure 2).

JuntionViewer was used to generate over 700 potential junction marker primers resulting in 35 junction markers that were used to map all 10 centromeres of maize. In addition, the visualization of gene homologies located within the repeat-rich centromeres helped us identify 15 single copy markers that we used to confirm the chromosome assignments of centromeric BAC sequences [15].

Next, we utilized JunctionViewer 2.0 to automatically visualize the centromeric features within several megabases of centromeric DNA sequence of the ZmB73v1 maize reference chromosomes [9,15]. For the custom annotation charts we created graphs using CENH3 associated reads that mapped uniquely, twice within one reference chromosome, or any number of times within the genome. We cut the reference chromosomes into 210 kb fragments that overlap by 10 kb, and used the parameters we had developed previously to visualize the major features within two centromeres. This enabled us to automatically obtain graphical representations of centromeres 2 and 5 as scalable PostScript images that were printed on poster paper to allow visualization of up to 10 Mb of contiguous centromere sequences [see Additional files 5 and 6].

Visualizing CentC and CRM arrangements in centromeres 2 and 5 revealed that CRMs in both centromeres frequently inserted into tandem CentC arrays. Expanded JunctionViewer displays of centromere 5 also illustrated a very distinct CRM1 and CRM2 distributions, as well as the presence of about one megabase of chromatin in the middle of centromere 5 that is free of CENH3. JunctionViewer images also facilitated the quantitation of 454 reads derived from chromatin immunoprecipitated with anti-CENH3 antibodies that mapped uniquely to CRM elements located within and outside of the functional centromeres [15].

Using MUMmer exact sequence match representations we were able to identify LTRs of retroelements with high probability, reducing the time to discover and date the insertions for these elements. As an example, we were able to identify two non-CRM retroelements that had inserted into CRM1 and CRM2 sequences based on MUMmer match and non-centromeric repeat homology depictions in JunctionViewer output [see Additional file 7]. The graphical representation of MUMmer results also revealed the presence of tandem repeats even where the sequence was unknown [see Additional file 8].

## Conclusions

Rapid and customizable annotation will become increasingly important as the rate of genome sequencing accelerates. JunctionViewer was developed, and is particularly suited, for automating the process of running, filtering, and graphically combining the best-fitting features of complex repeat sequences. It automatically merges various types of annotations across numerous and large regions of genomic DNA, and generates concise graphical representations of sequences, ranging in length from less than 1 kb to multiple megabases, that can be interpreted quickly. The concise graphical representations generated by JunctionViewer were critical for gaining insights about the sequence organization of maize centromeres. This software was designed to visualize key features of maize centromere sequences; however scientists may find it useful for annotating repeat-rich regions in other organisms. Another potential application of this software is the display of other sequence coverage data, e.g., illustration of the coverage of bacterial genome assemblies by short read shotgun sequences together with competed gene features to detect horizontal gene transfer.

## Availability and requirements

• **Project name: **JunctionViewer

• **Project home page: **http://junctionviewer.sourceforge.net/

• **Operating system(s): **Platform independent

• **Programming language: **Perl

• **Other requirements: **BioPerl, Perl Tk, NCBI BLAST and/or WU-BLAST, MUMmer, cross_match

• **License: **GNU GPLv3

• **Any restrictions to use by non-academics: **GNU GPLv3

## List of abbreviations used

BAC: bacterial artificial chromosome; CDS: coding sequence; CENH3: centromeric histone H3 variant; CentA: centromeric retrotransposon of maize similar to CRM3; CentC: centromeric tandem repeat of maize; ChIP: chromatin-immunoprecipitation; CRM: centromeric retrotransposon of maize; FPC: FingerPrinted Contigs; HSP: high-scoring segment pairs of a BLAST result; LTR: long terminal repeat of a retrotransposon; MGSC: Maize Genome Sequencing Consortium; OMA: oat-maize addition; PCR: polymerase chain reaction; TIGR: The Institute for Genomic Research; UTR: untranslated region of a retrotransposon.

## Authors' contributions

The software was conceptualized by TKW and GGP, and written by TKW. This manuscript was written and approved by TKW and GGP.

## Supplementary Material

Additional file 1 **JunctionViewer 2.0 package**. The gzipped tar (tarball) contains the JuntionViewer 2.0 package including the main program, documentation, and example files. Please see http://junctionviewer.sourceforge.net/ for the latest version. Click here for file

Additional file 2 **JunctionViewer 2.0 setup and usage**. JunctionViewer 2.0 (JV) can be set up and used in four steps: 1) install supporting software which enables JV to run, 2) define parameters for testing on a set of characterized sequences, 3) refine parameters until output matches what is expected for the characterized sequences, and 4) edit the refined parameters file to automatically process uncharacterized query sequences. Click here for file

Additional file 3 **JunctionViewer 1.0 display of a BAC sequence assembly fragment**. This JunctionViewer 1.0 display of a 3,393 nt BAC sequence assembly fragment includes graphical overlays (not created by JunctionViewer) indicating the type of sequences represented and where primers were designed. "*Zea *repeats" means TIGR Zea Repeats v3.0 database [20]. Cross_match and BLAST results show the similarities between CentA and CRM3 LTRs, which both align along the central part of the fragment. The difference is great enough, however, that this region can clearly be assigned to CentA. In this case, the unique junction sites were found between CRM2 and CentA. Click here for file

Additional file 4 **List of annotated sequences**. This table lists centromere features that are represented graphically in JunctionViewer 2.0 images of maize sequences, as well as a description of the sequence files used to detect each feature. Click here for file

Additional file 5 **Centromere 2**. Graphical representations are drawn for 210 kb regions overlapping 10 kb along 4.2 Mb of ZmB73v1 reference chromosome 2 (positions 88,190,001-92,400,000) including the centromeric and pericentromeric sequences. Several kb of plastid (purple) sequence appears at the edge of the centromere. The central regions include a complex mixture of CentC tandem repeat arrays (green filled arrows) interrupted by various repeated sequences (grey, maroon, blue, and tan). Notably, CRM2 and CRM1 elements (bounded by maroon LTRs and blue LTRs, respectively) dominate this section of the genome. Additionally, numerous nested retrotransposon insertions appear to have had a deleterious effect at least on the contiguity of CentC arrays. Click here for file

Additional file 6 **Centromere 5**. Graphical representations are drawn for two 210 kb regions overlapping by 10 kb along the central CentC region of centromere 5 (ZmB73v1 reference chromosome 5 positions 104,790,001-105,000,000 and 104,990,001-105,200,000). As in centromere 2, CentC and CRM sequences are relatively abundant and have been subjected to numerous insertions. Click here for file

Additional file 7 **JunctionViewer 2.0 display of nested retrotransposon insertions in the interstitial region of centromere 5**. The presence of non-CRM LTR retrotransposons and order of insertions are apparent and were confirmed by the dating method of SanMiguel et al. [10]: first a CRM1 element inserted into the genome (Kimura 2-parameter distance [κ]= 0.0346), followed by a CRM2 (κ = 0.0320), then the left non-CRM LTR retrotransposon (κ = 0.0175) inserted into the CRM1, and finally the non-CRM LTR retrotransposon (κ = 0.0017) jumped into the CRM2 sequence. This order of nested insertions is in accord with MUMmer sequence match representations, where longer match lines between LTRs correlate with younger elements. Click here for file

Additional file 8 **JunctionViewer 2.0 reveals the presence of a tandem repeat**. The foreground chart plots ChIP read match coverage results by BLAST (purple) [9]. This image displays the plots created using uniquely matched reads (red) and reads matching any number of times in the genome (grey). The stack of 10 grey vertical bars on the left of the display indicates the presence of a reference chromosome gap (1,000 Ns in the sequence). To the right is a smaller assembly gap (100 Ns) represented by one grey vertical bar. Between the gaps, red arrows at the bottom represent >= 100 nt exact sequence matches as reported by MUMmer. These arrows indicate the presence of a tandemly repeated sequence that was subsequently confirmed to be maize knob tandem repeat. Click here for file
