# Supplementary material for: JunctionViewer: customizable annotation software for repeat-rich genomic regions
Source: BMC Bioinformatics. 2010 Jan 12;11:23. doi: 10.1186/1471-2105-11-23 (PMC2824676; doi:10.1186/1471-2105-11-23)
Supplement: Additional file 2 — JunctionViewer 2.0 setup and usage. JunctionViewer 2.0 (JV) can be set up and used in four steps: 1) install supporting software which enables JV to run, 2) define parameters for testing on a set of characterized sequences, 3) refine parameters until output matches what is expected for the characterized sequences, and 4) edit the refined parameters file to automatically process uncharacterized query sequences. [file 1471-2105-11-23-S2.PDF]

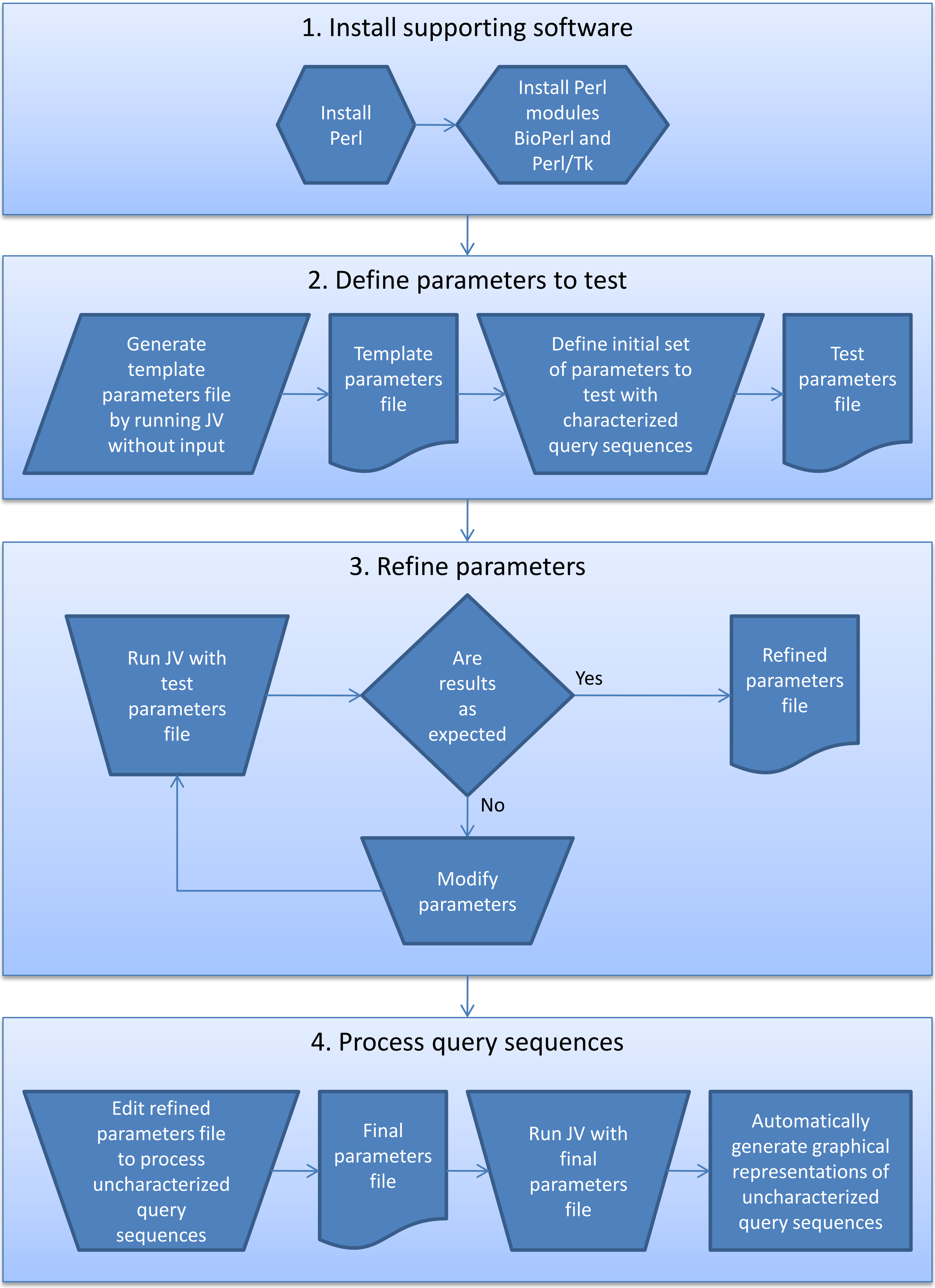
1. Install supporting software
Install Perl
Install Perl modules BioPerl and Perl/Tk
2. Define parameters to test
Generate template parameters file by running JV without input
Template parameters file
Define initial set of parameters to test with characterized query sequences
Test parameters file
3. Refine parameters
Run JV with test parameters file
Are results as expected
Yes
Refined parameters file
No
Modify parameters
4. Process query sequences
Edit refined parameters file to process uncharacterized query sequences
Final parameters file
Run JV with final parameters file
Automatically generate graphical representations of uncharacterized query sequences
