# Supplementary material for: JunctionViewer: customizable annotation software for repeat-rich genomic regions
Source: BMC Bioinformatics. 2010 Jan 12;11:23. doi: 10.1186/1471-2105-11-23 (PMC2824676; doi:10.1186/1471-2105-11-23)
Supplement: Additional file 3 — JunctionViewer 1.0 display of a BAC sequence assembly fragment. This JunctionViewer 1.0 display of a 3,393 nt BAC sequence assembly fragment includes graphical overlays (not created by JunctionViewer) indicating the type of sequences represented and where primers were designed. "Zea repeats" means TIGR Zea Repeats v3.0 database [20]. Cross_match and BLAST results show the similarities between CentA and CRM3 LTRs, which both align along the central part of the fragment. The difference is great enough, however, that this region can clearly be assigned to CentA. In this case, the unique junction sites were found between CRM2 and CentA. [file 1471-2105-11-23-S3.PDF]

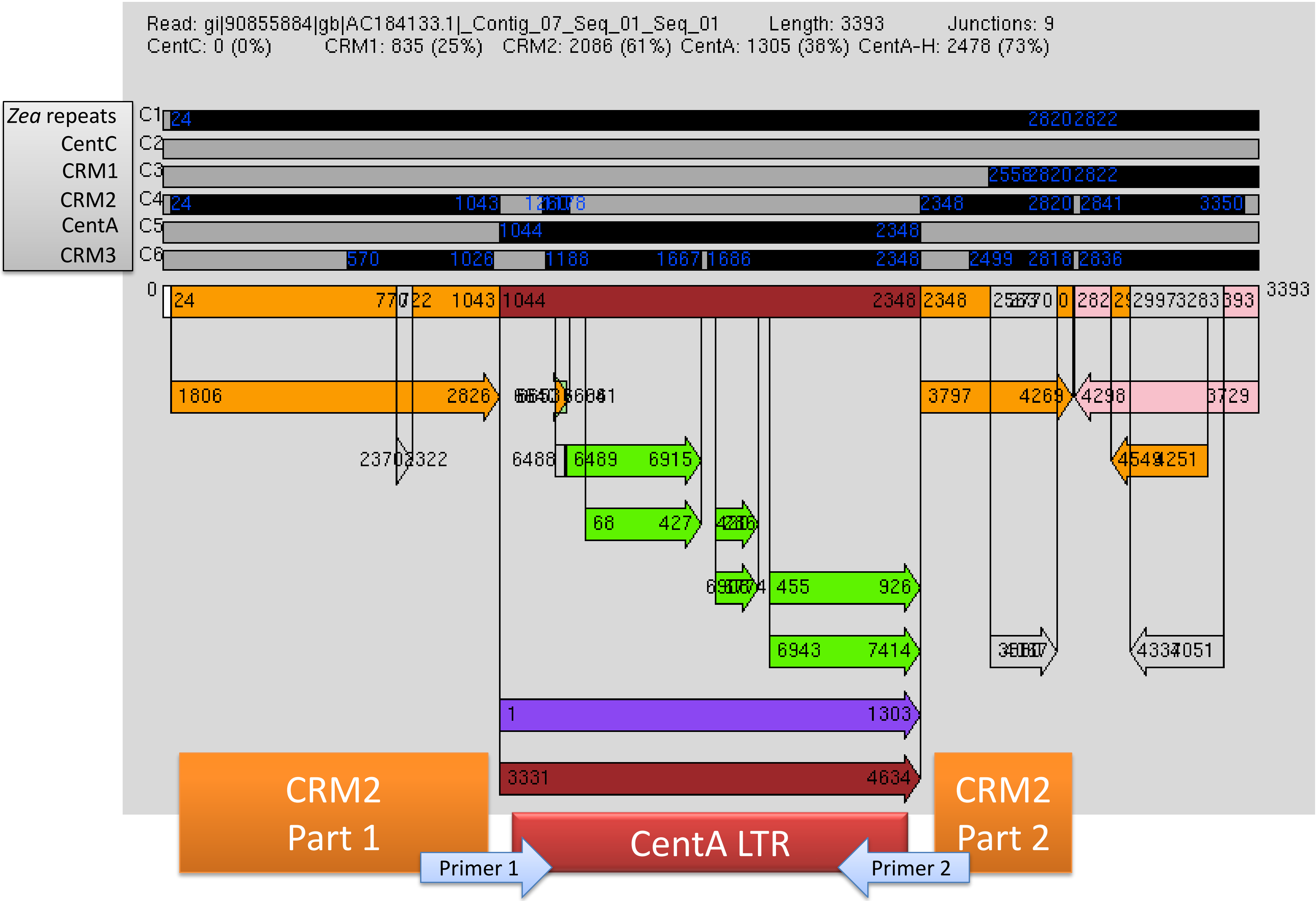
Read: gi|90855884|gb|AC184133.1|_Contig_07_Seq_01_Seq_01 Length: 3393 Junctions: 9
CentC: 0 (0%) CRM1: 835 (25%) CRM2: 2086 (61%) CentA: 1305 (38%) CentA-H: 2478 (73%)
Zea repeats
CentC
CRM1
CRM2
CentA
CRM3
C1
C2
C3
C4
C5
C6
CRM2 Part 1
CentA LTR
CRM2 Part 2
Primer 1
Primer 2
