# Supplementary material for: JunctionViewer: customizable annotation software for repeat-rich genomic regions
Source: BMC Bioinformatics. 2010 Jan 12;11:23. doi: 10.1186/1471-2105-11-23 (PMC2824676; doi:10.1186/1471-2105-11-23)
Supplement: Additional file 4 — List of annotated sequences. This table lists centromere features that are represented graphically in JunctionViewer 2.0 images of maize sequences, as well as a description of the sequence files used to detect each feature. [file 1471-2105-11-23-S4.DOC]

| JunctionViewer 2.0 annotated sequences. | |
| --- | --- |
| Name | Description |
| CRM1 A LTR | One consensus sequence [8]. |
| CRM1 R4/R5 LTR | Two consensus sequences (AB and AB with a deletion) [8]. |
| CRM1 R3 LTR | One consensus sequence [8]. |
| CRM1 R2 LTR | One consensus sequence [8]. |
| CRM1 B/R1 LTR | Seven consensus sequences (4 short and 3 long) [8]. |
| CRM2 LTR | One consensus sequence [4]. |
| CRM3 LTR | One consensus sequence [4]. |
| CRM1/2/3 CDS | Seven consensus sequences (5 CRM1, 1 CRM2, and 1 CRM3) [4]. |
| CentA LTR/CDS | Two consensus sequences (LTR and CDS) [1]. |
| CentC | One consensus sequence from 9674 monomers in BACs sequenced by the Maize Genome Sequencing Consortium [9]. |
| Maize repeats | TIGR Zea Repeats v3.0 database with centromeric sequences CentC/CRM masked by cross_match [20]. |
| Maize genes | A total of 1,266 mRNA sequence records remaining from the *Zea mays* RefSeq portion of GenBank downloaded on 4 February 2008 after removing 1 transposase (NM_001111483.1) and 4 organellar (NC_001400.1, NC_001666.2, NC_007982.1, and NC_008332.1) records. |
| Rice genes | A total of 51,285 coding sequence records remaining from version 5.0 of the Rice Genome Annotation Project [http://rice.plantbiology.msu.edu/] after removing 15,425 records with the substring "[T/t]ranspos" in their descriptions. |
| Maize organelle | A total of 4 chloroplast/mitochondrial sequence records (NC_001400.1, NC_001666.2, NC_007982.1, and NC_008332.1) downloaded from the *Zea mays* RefSeq portion of GenBank on 4 February 2008. |
