# Supplementary figures and images for: JunctionViewer: customizable annotation software for repeat-rich genomic regions
Source: BMC Bioinformatics. 2010 Jan 12;11:23. doi: 10.1186/1471-2105-11-23 (PMC2824676; doi:10.1186/1471-2105-11-23)

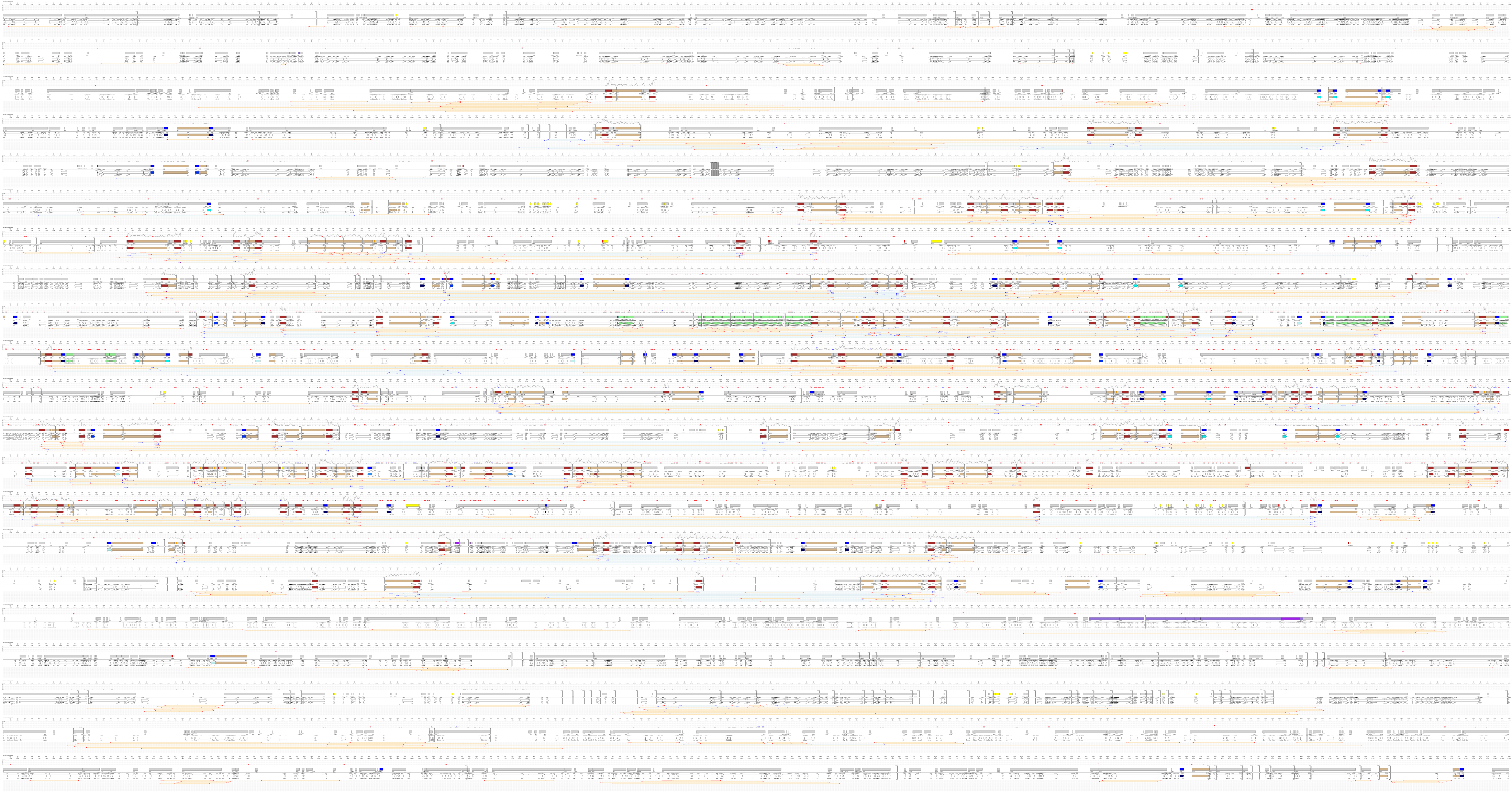

Supplement: Additional file 5 — Centromere 2. Graphical representations are drawn for 210 kb regions overlapping 10 kb along 4.2 Mb of ZmB73v1 reference chromosome 2 (positions 88,190,001-92,400,000) including the centromeric and pericentromeric sequences. Several kb of plastid (purple) sequence appears at the edge of the centromere. The central regions include a complex mixture of CentC tandem repeat arrays (green filled arrows) interrupted by various repeated sequences (grey, maroon, blue, and tan). Notably, CRM2 and CRM1 elements (bounded by maroon LTRs and blue LTRs, respectively) dominate this section of the genome. Additionally, numerous nested retrotransposon insertions appear to have had a deleterious effect at least on the contiguity of CentC arrays. [file 1471-2105-11-23-S5.PDF]

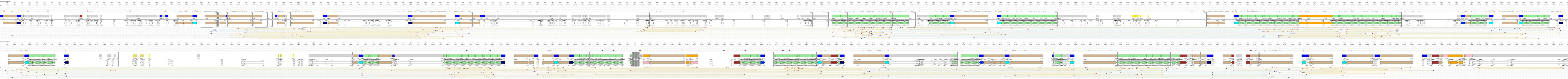

Supplement: Additional file 6 — Centromere 5. Graphical representations are drawn for two 210 kb regions overlapping by 10 kb along the central CentC region of centromere 5 (ZmB73v1 reference chromosome 5 positions 104,790,001-105,000,000 and 104,990,001-105,200,000). As in centromere 2, CentC and CRM sequences are relatively abundant and have been subjected to numerous insertions. [file 1471-2105-11-23-S6.PDF]

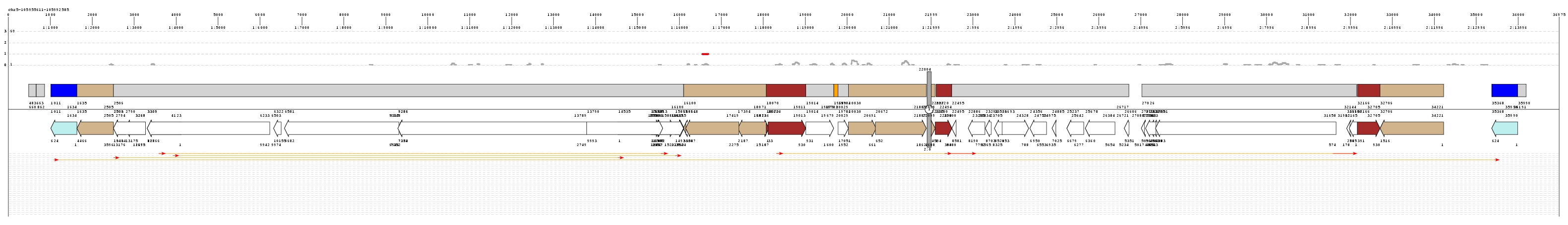

Supplement: Additional file 7 — JunctionViewer 2.0 display of nested retrotransposon insertions in the interstitial region of centromere 5. The presence of non-CRM LTR retrotransposons and order of insertions are apparent and were confirmed by the dating method of SanMiguel et al. [10]: first a CRM1 element inserted into the genome (Kimura 2-parameter distance [κ]= 0.0346), followed by a CRM2 (κ = 0.0320), then the left non-CRM LTR retrotransposon (κ = 0.0175) inserted into the CRM1, and finally the non-CRM LTR retrotransposon (κ = 0.0017) jumped into the CRM2 sequence. This order of nested insertions is in accord with MUMmer sequence match representations, where longer match lines between LTRs correlate with younger elements. [file 1471-2105-11-23-S7.JPEG]

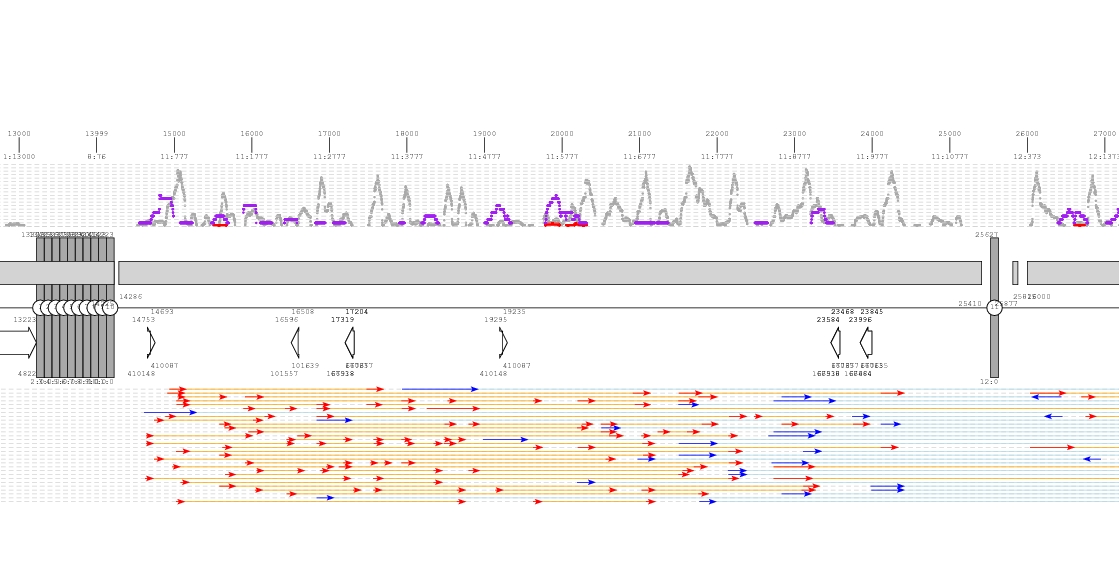

Supplement: Additional file 8 — JunctionViewer 2.0 reveals the presence of a tandem repeat. The foreground chart plots ChIP read match coverage results by BLAST (purple) [9]. This image displays the plots created using uniquely matched reads (red) and reads matching any number of times in the genome (grey). The stack of 10 grey vertical bars on the left of the display indicates the presence of a reference chromosome gap (1,000 Ns in the sequence). To the right is a smaller assembly gap (100 Ns) represented by one grey vertical bar. Between the gaps, red arrows at the bottom represent >= 100 nt exact sequence matches as reported by MUMmer. These arrows indicate the presence of a tandemly repeated sequence that was subsequently confirmed to be maize knob tandem repeat. [file 1471-2105-11-23-S8.JPEG]
